# Anatomy for right ventricular lead implantation

**DOI:** 10.1007/s00399-022-00872-w

**Published:** 2022-06-28

**Authors:** Carsten W. Israel, Sona Tribunyan, S. Yen Ho, José A. Cabrera

**Affiliations:** 1 Klinik für Innere Medizin—Kardiologie, Diabetologie & Nephrologie, Evangelisches Klinikum Bethel, Burgsteig 13, 33617 Bielefeld, Germany; 2 Cardiac Morphology Unit, Royal Brompton and Harefield NHS Foundation Trust and Imperial College London, SW3 6NP London, UK; 3 Unidad de Arritmias, Departamento de Cardiología, Hospital Universitario Quirón-Salud Madrid and Complejo Hospitalario Ruber Juan Bravo, Universidad Europea de Madrid, Madrid, Spain

**Keywords:** Right ventricular outflow tract, Septal right ventricular pacing, Septomarginal trabeculations, Fluoroscopy, Moderator band, Rechtsventrikulärer Ausflusstrakt, Septale rechtsventrikuläre Stimulation, Septomarginale Trabekel, Fluoroskopie, Moderatorband

## Abstract

To understand the position of a pacing lead in the right ventricle and to correctly interpret fluoroscopy and intracardiac signals, good anatomical knowledge is required. The right ventricle can be separated into an inlet, an outlet, and an apical compartment. The inlet and outlet are separated by the septomarginal trabeculae, while the apex is situated below the moderator band. A lead position in the right ventricular apex is less desirable, last but not least due to the thin myocardial wall. Many leads supposed to be implanted in the apex are in fact fixed rather within the trabeculae in the inlet, which are sometimes difficult to pass. In the right ventricular outflow tract (RVOT), the free wall is easier to reach than the septal due to the fact that the RVOT wraps around the septum. A mid-septal position close to the moderator band is relatively simple to achieve and due to the vicinity of the right bundle branch may produce a narrower paced QRS complex. Special and detailed knowledge is necessary for His bundle and left bundle branch pacing.

## Abstract (de)

Um die Position einer Schrittmacherelektrode im rechten Ventrikel zu verstehen und Fluoroskopie sowie intrakardiale Signale richtig interpretieren zu können, sind gute anatomische Kenntnisse erforderlich. Der rechte Ventrikel kann in einen Einflusstrakt, einen Ausflusstrakt und einen apikalen Bereich unterteilt werden. Ein- und Ausflusstrakt werden durch die septomarginalen Trabekel voneinander getrennt, der apikale Bereich liegt unterhalb des Moderatorbands. Eine Elektrodenlage im rechtsventrikulären Apex ist nicht zuletzt aufgrund des dort dünnen Myokards nicht günstig und oft gar nicht einfach zu erreichen; die Elektrodenspitze bleibt leicht in den Trabekeln des Einflusstrakts hängen, die manchmal schwer zu passieren sind. Im rechtsventrikulären Ausflusstrakt (RVOT) ist die freie Wand einfacher zu erreichen als die septale, da der RVOT sich um das Septum herumwindet. Eine mittseptale Position nahe dem Moderatorband ist relativ leicht zu erreichen und erzielt durch die Nähe zum rechten Schenkel oft einen schmaleren stimulierten QRS-Komplex. Spezielle und detaillierte anatomische Kenntnisse sind für eine His-Bündel- und Linksschenkelstimulation erforderlich.

Endocardial right ventricular (RV) pacing leads have been implanted since the early 1960s. Traditionally, the site for implantation of the RV lead tip is the RV apex since it is easy to reach from the superior vena cava and offers lead stability, allowing the implantation of passive-fixation leads. However, in many patients the wall of the RV apex is relatively thin and perforation is not an infrequent complication (risk of perforation 3.4-fold for RV apical pacing, [6]). Even more importantly, RV apical pacing is usually associated with very wide QRS complexes. Since the advent of cardiac resynchronization therapy, a wide QRS duration in the context of left bundle branch block morphology has been associated with ventricular desynchronization, deterioration of ventricular function, and adverse ventricular remodeling.

Numerous studies have been performed in the last two decades to investigate whether a non-apical RV lead position offers any advantage over apical RV pacing, e.g., concerning mortality, the development of heart failure, quality of life, exercise capacity, etc. These studies have had different results (positive and neutral) depending on endpoints, selected patients, and study duration. Additionally, in most studies the exact location of the “alternative” RV pacing site was not well defined. Despite a meta-analysis showing an advantage of RV septal versus RV apical pacing on echocardiographic parameters, the majority of the authors of the current European guidelines on cardiac pacing felt that the evidence was not sufficient to support a recommendation to systematically implant RV leads at the septum instead of the apex [8]. Only for elderly and female patients and those with a body mass index < 20 kg/m^2^, RV septal pacing is recommended to avoid perforation. However, implantation habits have changed considerably in Germany over the last two decades: 20 years ago, more than 90% of RV leads were implanted in the RV apex; today, the majority of RV leads are implanted at the mid RV septum. And although the authors of the European guidelines on pacing and the authors of the European consensus statement on device implantation [1] did not give a recommendation on any first-line RV pacing site, personally all of them would rather implant the RV lead at the septum than in the RV apex.

One of the limitations to the use of RV pacing sites other than the RV apex may be suboptimal knowledge about RV anatomy, particularly how to reach and use sites with good conditions for lead stability, low risk of complications, and electrical or hemodynamic advantages. Highly detailed knowledge about RV anatomy becomes even much more important in light of conduction system pacing, i.e., His bundle and left bundle branch pacing. Some of the information about RV anatomy is presented in this overview, particularly by anatomic and fluoroscopic pictures.

## Anatomy of the RV

The RV is located anteriorly behind the sternum and marks the lower border of the cardiac silhouette but usually not the apical border, which is marked by the left ventricular (LV) apex. The RV apex is frequently inferior to the LV apex.

**Fig. 1:**
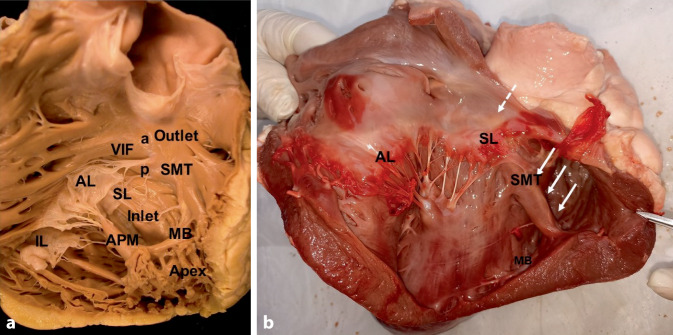
Anatomy of the right ventricle (RV).** a** In a frontal view, the tricuspid valve is seen with its septal leaflet (*SL*), anterior leaflet (*AL*), and inferior leaflet (*IL*). They lead to the inlet compartment of the RV. At the border to the outlet compartment, there is the septomarginal trabeculation (*SMT*) with an anterior (*a*), superior, and a posterior (*p*), inferior branch. At the septum, outlet and inlet are connected by the ventriculo-infundibular fold (*VIF*). The moderator band (*MB*) crosses the RV from SMT to the anterior papillary muscle (*APM*) and marks the border between inlet and apical compartment of the RV. With permission © Sieuw Yen Ho, London.** b** View to the inlet compartment of the RV (bovine heart). Note the fasciculae of the right bundle branch (*arrows*) within the septomarginal trabeculation (SMT) and moderator band (*MB*). The atrioventricular node and atrial part of the proximal His bundle (*dotted arrows*) are shining through the endocardium above the septal leaflet (*SL*) of the tricuspid valve. *AL* anterior leaflet. Note the thinning of the myocardium at the RV apex

Viewed from the front, the RV has a triangular shape with a horizontal lower line from the tricuspid valve to the apex, a perpendicular line from the apex to the pulmonary valve, and a third line from the pulmonary valve back to the tricuspid valve. This “line” from the pulmonary to the tricuspid valve is not straight but impressed by a muscular fold, the ventriculo-infundibular fold (VIF). The VIF marks the border between the inlet and outlet of the RV and consists of: (i) a parietal band, (ii) a subpulmonary conus that transitions into the outlet septum, (iii) the septal band, and (iv) the septomarginal trabeculation (Fig. 1a). The septomarginal trabeculation inserts at its septal (proximal) margin with an anterior(-superior) and a posterior(-inferior) limb, forms the supraventricular crest and, at its distal margin, transitions into the moderator band (MB). The MB crosses the RV cavity to the anterior papillary muscle. It marks the border between inlet and apical compartments of the RV.

The septal (or medial) papillary muscle inserts into the posterior arm of the septomarginal trabeculation, while the anterior arm of the septomarginal trabeculation transitions into the subpulmonary infundibulum. The proximal part of the septomarginal trabeculation with the anterior and posterior arm is not always attached to the septum, and can be hypertrophied and impede access to the right ventricular outflow tract (RVOT).

The take-off of the right bundle branch of the conduction system is typically located at the insertion of the medial papillary muscle and leads to the MB, which originates between the anterior and posterior arm of the septomarginal trabeculation and crosses the RV to the parietal wall. It carries a fascicle of the right bundle branch (Fig. 1b).

The muscular trabeculations in the RV apex are coarser than those in the LV. The wall at the very tip of the apex is thin, often < 1 mm (Fig. 1). Apart from a small fibrous portion—the membranous septum—the ventricular septum is muscular.

The inlet of the RV extends from the tricuspid valve plane to the insertions of the papillary muscles into the ventricular walls. The tricuspid valve has three leaflets; however, the divisions between the leaflets are sometimes not easy to discriminate. The septal leaflet has multiple tendinous cords attached to the ventricular septum. At its antero-superior part, it divides the membranous septum into atrioventricular (AV) and interventricular portions. The septal papillary muscle is normally the least prominent of all RV papillary muscles and can frequently not be distinguished from the ventricular wall. The anterior leaflet is large and caudal to the septal leaflet. The anterior papillary muscle is usually the largest, leading to the anterior free wall and connected to the moderator band close to its insertion at the parietal wall. The inferior leaflet, also called posterior or mural, is connected to the inferior, diaphragmatic wall of the RV by one or more small papillary muscles.

The RV overlaps and crosses the LV; the RVOT wraps around the LV, the high RVOT being left and above of the LV outflow tract (LVOT). The LVOT and aortic root are wedged behind the septum between the RV inlet and outlet (Fig. 3).

The VIF stretches from the septum above the tricuspid valve to the subpulmonary infundibulum of the RVOT, the muscular tube below the pulmonary valve and above the ventricular septum. The anterosuperior wall of the RV completes this muscular tube. The subpulmonary infundibulum is usually free of muscular trabeculations (Fig. 3). However, trabeculations of variable size and prominence usually run at the border between septal and parietal free wall from the apex to the subpulmonary recess.

The normal muscular RV wall (not including trabeculations) is 3–5 mm thick. Myocardial fiber orientation is radial at superficial layers and longitudinal from the apex to the base at inner layers. The subepicardial myofibers run parallel to the AV groove, encircling the subpulmonary infundibulum. At the RV apex, superficial myofibers invaginate the subendocardial myofibers spirally.

## Fluoroscopy for assessing RV lead position

**Fig. 2:**
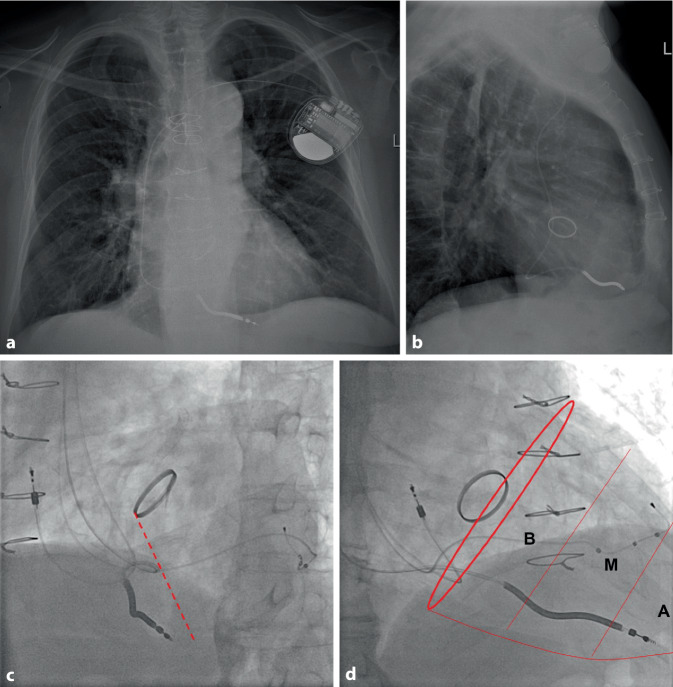
Dual-chamber implantable cardioverter-defibrillator (ICD) with a lead position in the right ventricular (RV) apex. Patient with sternotomy and aortic valve replacement. **a** Chest X‑ray in postero-anterior (PA) projection.** b** Chest X‑ray in right lateral 90° projection. The ventricular lead is implanted in the RV apex. Since this is an ICD lead (stiffer than a pacemaker lead), perforation at the RV apex is a risk, particularly if abundant lead slack (big loop in the right atrium in the lateral projection!) pushes the lead against the RV apex with each heartbeat.** c** Left anterior oblique (LAO) 40° view (after upgrade to a biventricular ICD). The RV lead positioned in the RV apex points towards 5 o’clock. *Dotted line*: approximate ventricular septum. **d** Right anterior oblique (RAO) 30° projection. The lead tip is positioned deep in the RV apex but still millimeters apart from the silhouette (otherwise: suspicion of perforation). *Red circle*: presumed approximate atrioventricular valve plane, the ventricular silhouette is divided into three segments: basal (*B*), mid (*M*), and apical (*A*) third

RV lead location is assessed by fluoroscopy during implantation, and after implantation usually by chest X‑ray using a posterior-anterior (PA) and a 90° lateral projection (Fig. 2a, b). While the PA view can reliably reflect the site of the lead tip in a caudo-cranial and a left–right axis, it cannot discriminate a posterior from an anterior implantation site. This information is best provided by left and right oblique projections (Fig. 2b–d). In the left anterior oblique (LAO) view with an angle of 40°, an RV lead at the mid-septum typically points towards 3 o’clock, a lead tip implanted in the high RV outflow tract to 2 o’clock, a lead in the RV apex to 5 o’clock. To understand whether an RV lead is implanted at the apex, in the middle third (mid-septal RV, distal left bundle branch) or rather at the base (RV inflow or outflow tract, His bundle, or proximal left bundle branch), fluoroscopy from the right anterior oblique (RAO) angle of 30° is usually optimal. At the end of implantation, these three projections (PA, LAO 40°, and RAO 30°, Fig. 2) should be used to document the final result at implantation.

## Implantation at the RV apex

The RV apex usually shows a high degree of trabecularization (Fig. 1a), which facilitates lead stabilization. It is the only site in the RV where a passive-fixation lead may be implanted [1]. However, shape and inner surface of the RV apex vary and in patients with RV congestion, the RV apex can become large, round, the trabeculae rather flat, and the wall rather thin (Fig. 1b). In some patients, it may be difficult to reach the true apex behind a number of trabeculae (Fig. 1a). In these cases, the lead may inadvertently be fixed at the posterior free wall of the RV inlet, comparatively close to the tricuspid wall. This position is not good for stability and chronic pacing threshold since movements of the tricuspid valve and deep respiration can pull on the lead. RV apical pacing is associated with ventricular desynchronization, adverse remodeling, and pacing-induced cardiomyopathy. Pacing at this site can cause late contraction of the proximal ventricular septum, compromising diastolic blood flow in the left artery descending. Therefore, most experienced implanters avoid this RV lead location.

## High RV outflow tract

**Fig. 3:**
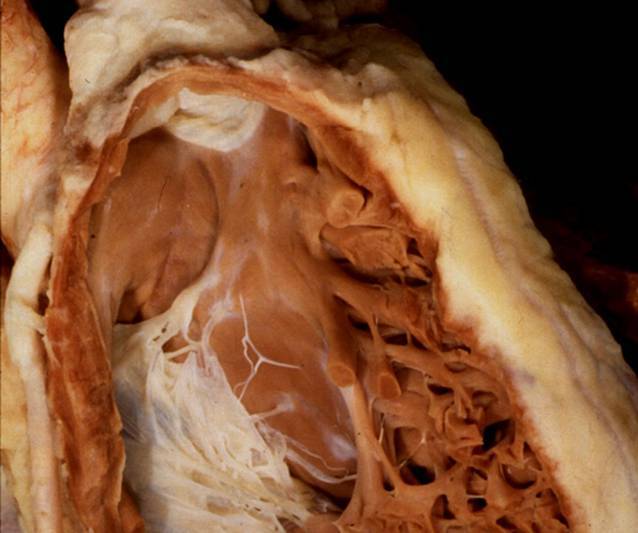
Right ventricular outflow tract. The medial septal part has a smooth surface, while the anterior part of the muscular tube has multiple trabeculae and recesses below the pulmonary valve. With permission, © Siew Yen Ho, London, UK

Lead implantation at the high RVOT can be identified in the 12-lead ECG by a completely negative QRS complex in lead I and a QRS axis at approximately 90°. If the lead has been fixed to the septal part of the RVOT, the paced QRS complex can be rather narrow (130–140 ms) with no or only minor notching. However, if the lead has been fixed at the anterior free wall of the high RVOT, the paced QRS complex can become very broad and notched. Lead fixation at the high septal RVOT is somewhat difficult since the inner surface at this site is usually smooth with few trabeculae (anterior septomarginal trabeculations, Fig. 3) and the angle between the inlet and outlet is comparatively sharp (Fig. 1a). It is much easier to fix the lead at the anterior high RVOT, which is usually highly trabeculated and offers one or more recesses below the pulmonary valve (Fig. 3) with very good long-term stability. However, between the trabeculae the anterior parietal wall of the high RVOT can be relatively thin and there is a risk of perforation or penetration.

## Mid-septal RV lead implantation

**Fig. 4:**
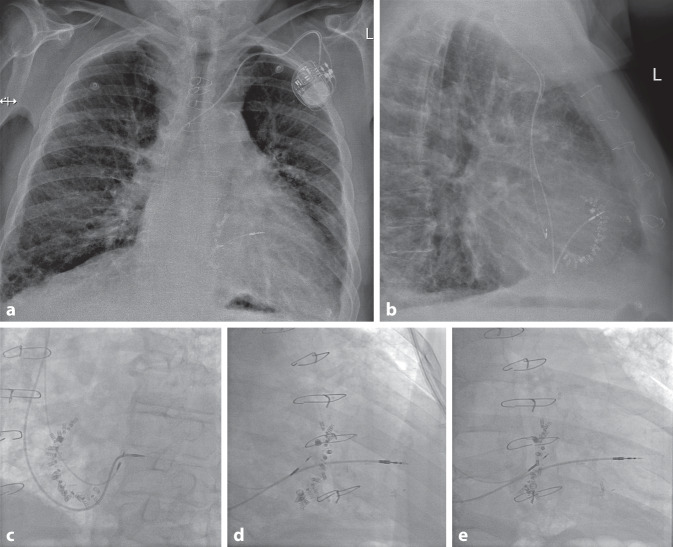
Right ventricular (RV) mid-septal and His bundle pacing in a patient with tricuspid valve repair. In this patient with a Cardioband® (Edwards, Irvive, CA, USA), the ring of the tricuspid valve is visible. **a** Chest X‑ray in the posterior-anterior view. The mid-septal RV back-up lead points towards the right side (3 o’clock), the bipolar tip of the thin His bundle pacing lead is attached on the ventricular part of the His bundle at the “roof” of the inlet compartment. **b** Chest X‑ray in the right lateral view (90°). The RV back-up lead points towards the sternum, overlapping the anterior end of the tricuspid valve. The His bundle lead is positioned close to the posterior end of the tricuspid ring. **c** Fluoroscopy in left anterior oblique (LAO) 40° projection. This is a fundamental view to understand the lead position. The RV back-up lead points straight to the spine (3 o’clock position). The His bundle lead is positioned just superior to the septal leaflet of the tricuspid valve. **d** Fluoroscopy in right anterior oblique (RAO) 30° projection. The RV back-up lead still points towards 3 o’clock, in this projection the middle third of the RV (basal, mid, apical). The His bundle lead seems to be on the atrial side of the tricuspid valve. **e** Fluoroscopy in RAO 12° projection. In this view, the leaflets of the tricuspid valve completely overlap; therefore the exact site of His bundle lead implantation can be assessed. The tip is obviously at the rather distal part, some millimeters into the ventricular side

A position at the mid-septal RV is the most interesting for ventricular pacing outside cardiac resynchronization therapy or conduction system pacing. On fluoroscopy, the lead tip points towards 3 o’clock in PA, LAO 40°, and RAO 30° projections (Fig. 4). In LAO 40° projection, it points rather horizontally to the spine, and in RAO 30° towards the wall in the middle third of the RV. Frequently, the true lead position is somewhat at the margin between the septal and parietal RV wall since the septal aspects of the mid RV are comparatively smooth, the target area small, and the angle sharp. However, fixation is sometimes easy near the moderator band, which can be confirmed by the ECG with a rather narrow paced QRS complex due to proximity of the pacing site to the right bundle within the moderator band (Fig. 1).

Lead implantation at the mid-septal RV requires the use of multiple fluoroscopic views and of a specially shaped stylet (with three curves, introduced by Harry Mond [9, 15]), as outlined in the European Heart Rhythm Association (EHRA) consensus paper [2]. Fluoroscopy cannot always clarify whether the lead tip is truly implanted at the septum or parietal at the anterior free wall.

## His bundle pacing

Lead implantation at the His bundle offers the advantage of pacing via the native conduction system including Tawara branches and Purkinje fibers, in a narrow intrinsic QRS complex with a maximum of ventricular synchrony. However, implanting the pacing lead at the proximal (atrial) or distal (ventricular) portion requires some anatomical knowledge.

**Fig. 5:**
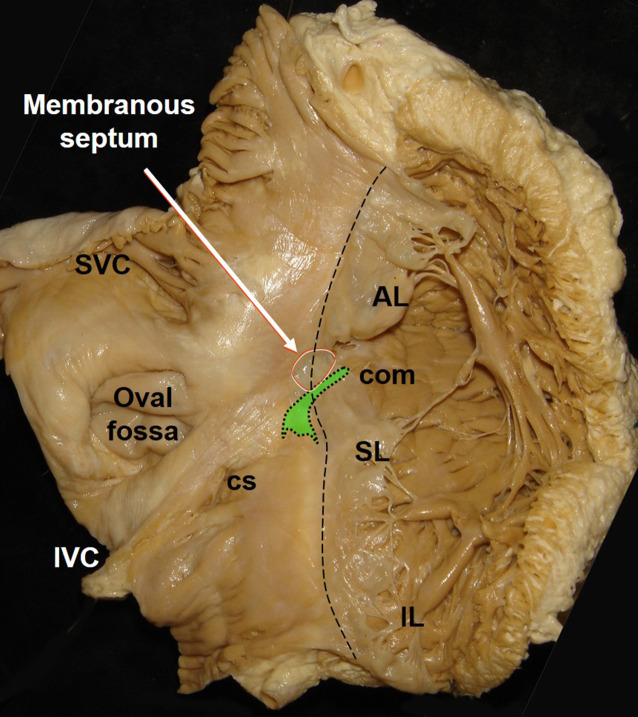
Atrioventricular (AV) node and His bundle. The AV node (*green with dotted points*) is located on the atrial side, slightly above the insertion of the septal leaflet of the tricuspid valve and anterior to the coronary sinus (*CS*) ostium. Its distal portion points towards the commissure (*com*) between the septal and anterior leaflet. It transitions to the His bundle on the atrial part of the membranous septum, which is separated from the ventricular membranous septum by the septal leaflet. *White circle* and *arrow*: membranous septum, *AL* anterior leaflet, *IL* inferior leaflet, *IVC* inferior vena cava, *SL* septal leaflet, *SVC* superior vena cava. With permission, © Siew Yen Ho, London, UK

**Fig. 6:**
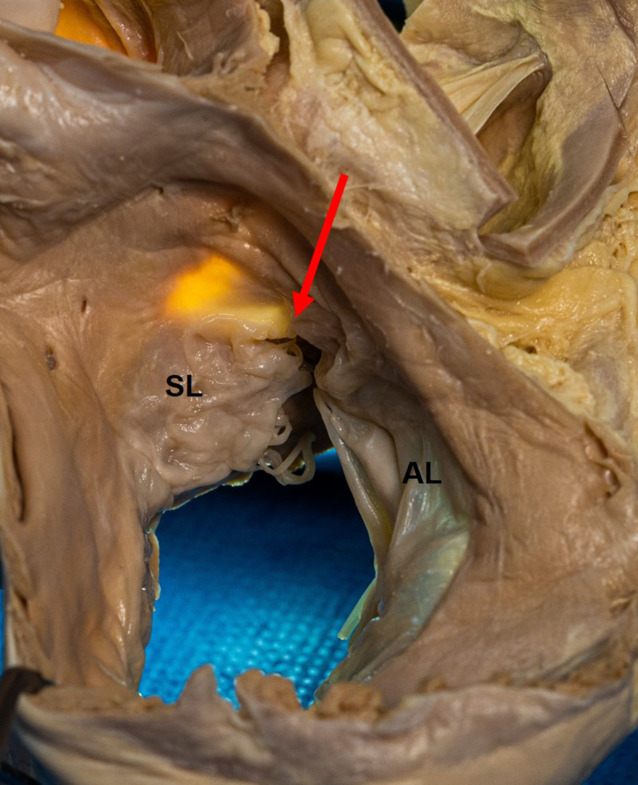
View from the right atrium to the tricuspid valve and entrance to the right ventricle. The His bundle typically perforates the tricuspid annulus at the commissure (*red arrow*) between septal (*SL*) and anterior leaflet (*AL*) of the tricuspid valve. The atrial and interventricular membranous septum is transilluminated. © With permission José Angel Cabrera, Madrid, Spain

A number of excellent reviews about the anatomy of His bundle pacing have recently been published [3, 7, 12–16]. The compact AV node is usually situated on the atrial side behind (posterior to) the septal leaflet of the tricuspid valve (Figs. 4 and 5) in the triangle of Koch, which is constituted by the tricuspid valve annulus on the right side, the tendon of Todaro at the left side, and an imaginary line at the coronary sinus ostium at the base. The atrial portion of the His bundle runs from the distal end of the AV node in a superior direction to the membranous septum (the upper tip of the triangle of Koch), which is divided by the septal leaflet into an atrial and a ventricular part (Figs. 5 and 6). Here, it perforates the tricuspid annulus, typically close to the antero-septal commissure (Figs. 5 and 6). In this area, the His bundle lies within connective tissue of the central fibrous body [12], insulated by a sheath of connective tissue. On the right side of the septum, the central fibrous body is next to the commissure between the septal and anterior leaflets of the tricuspid valve. Opposite on the left side of the septum, it is next to the commissure of the right and non-coronary cusps of the aortic valve. At this site, there is no myocardial tissue surrounding the His bundle, neither atrial nor ventricular muscle. Implanting a His bundle pacing lead at this exact spot will likely result in no undesired atrial sensing, excellent selective His bundle stimulation, but frequently poor ventricular sensing (R wave typically < 2.0 mV and often < 1.0 mV), and a variable, often high pacing threshold. If the lead is moved from this spot at the tip of the triangle of Koch a little more inferior and backwards to the proximal His bundle (Fig. 5), P wave (farfield) sensing occurs due to the close atrial myocardial layer. Not infrequently, there is seemingly selective His bundle pacing at this site, but if His bundle capture is lost, atrial capture is dismantled. If the lead is moved a little forward through the commissure between the septal and anterior leaflet of the tricuspid valve and a little superior, the more distal part of the His bundle at the ventricular part of the membranous septum can be reached. This part of the more distal His bundle at the membranous septum can be reached antegrade as well as retrograde by pulling back to the commissure between the septal and anterior leaflets of the tricuspid valve. Immediately after penetrating the tricuspid valve annulus, the His bundle runs superficially through the membranous septum on the muscular crest of the interventricular septum, surrounded only by connective tissue. At this point, still selective His bundle capture can be expected with some better (“farfield”) sensing (e.g. 2.0–4.0 mV) due to the closer proximity to the ventricular myocardium. After a variable length, the His bundle perforates the septum, is covered by a layer of ventricular myocardium of variable thickness, and then divides into the left and right Tawara branches. This division can be superficial on the right side of the septum, deep in the muscular septum, and even on the left side subendocardially. On the right side, the right bundle branch typically emerges at the base of the septal papillary muscle and continues with a large branch to and within the moderator band.

Interestingly, the His bundle has a fibrous insulation and is a palpable, hard, chord-like structure. It varies up to 20 mm in length and 4 mm in diameter [10–12]. The proximal bundle branches are also insulated by connective tissue that is a direct continuation of the fibrous body [12]. This explains the isoelectrical line without “pseudodelta wave” between stimulus and beginning of the QRS complex in selective His bundle pacing, typically with a duration of approximately 40 ms. The more distal the pacing lead is implanted, the more likely the ventricular myocardium is captured, resulting in non-selective His bundle pacing with a “pseudodelta wave” between stimulus and sharp deflection of the R wave. The size and width of this pseudodelta wave depends on the His bundle and myocardial thresholds; a high His bundle threshold as well as a low septal myocardial threshold at this site can cause a large pseudodelta wave.

## Left bundle branch pacing

**Fig. 7:**
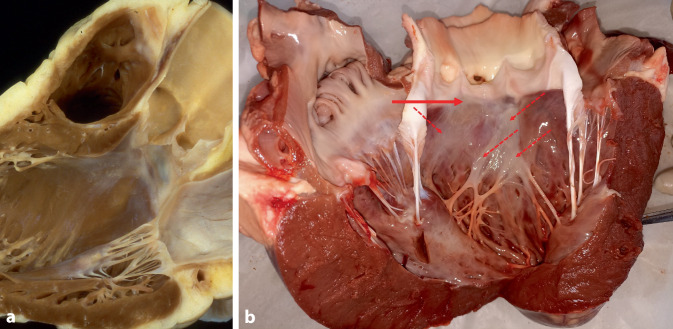
View on the subendocardial branches of the left bundle branch (LBB). The LBB penetrates the membranous septum between the right and the non-coronary cusps of the aortic valve and divides into a widespread subendocardial network on the left side (white subendocardial fibers). **a** Human heart, with permission, © Siew Yen Ho, London, UK. **b** Bovine heart. *Solid arrows*: location of transseptal perforation of the LBB between the non-coronary and right coronary cusps; *dotted arrows*: widespread network of multiple subendocardial fascicles of the LBB

The left bundle branch (LBB) can be accessed for direct pacing from the right side of the septum after division of the His bundle into Tawara branches and before it penetrates the septum. However, this spot is very small, variable in location, and difficult to find. It is much easier to pace the more distal part of the LBB trunk that later divides into a widespread subendocardial network on the left side (Fig. 7). This requires transseptal implantation of a thin screw-in lead that has to go all the way from the right side to the very subendocardial tissue on the left side. The left ventricular subendocardial branches of the LBB usually extend from the inferior border of the membranous septum to the crest of the muscular ventricular septum. After penetrating the membranous septum, the LBB has a nonbranching section of 1–3 mm in length along the septal crest before dividing into the fascicles on the septal surface (Fig. 7).

From the left side, the membranous septum is divided into an AV and an interventricular component at the base of the interleaflet triangle between the right and the non-coronary leaflets of the aortic valve [4, 5]. The length of the membranous septum is variable, typically approximately 5 mm with a range of 1–9 mm [4, 5]. Frequently, the interventricular part of the membranous septum is small and shows an early division of the fascicles of the LBB already at the level of the hinge of the septal leaflet of the tricuspid valve.

LBB anatomy has marked variability. The LBB origin can be broad or narrow (< 1 to 14 mm). Distally, it becomes broader, abruptly or gradually, with a variable size, number, location, configuration, and distribution of branches. Despite all variability, three endocardial main networks of the LBB can be distinguished, a thin elongated anterior bundle of branches (left anterior fascicle), a wider posterior bundle of branches (left posterior fascicle), and a third bundle of branches that covers the left ventricular mid-septal surface (left septal fascicle). The anterior-superior fascicle is often thin and long and extends towards the superolateral papillary muscle. The infero-posterior fascicle is usually thicker, broader, and shorter and runs towards the base of the inferoseptal papillary muscle. The septal branch emerges between the infero-posterior and antero-superior fascicles. The main LBB trunk before its division may be the best target for pacing. It extends 10–15 mm in an inferior direction towards the apex. Therefore, implanting a pacing lead approximately 1.0–1.5 cm below the tricuspid annulus through the mid-septal area may have the highest chance of success. However, due to the high anatomic variability, it is not predictable if a branch, e.g. septal, rather than the main LBB trunk is reached when screwing the lead into the septum from the right side. On fluoroscopy, a RAO view at 30° with the lead pointing towards a 2 o’clock position is usually optimal to assess a good starting point for implantation of a transseptal LBB pacing lead.

**Fig. 8:**
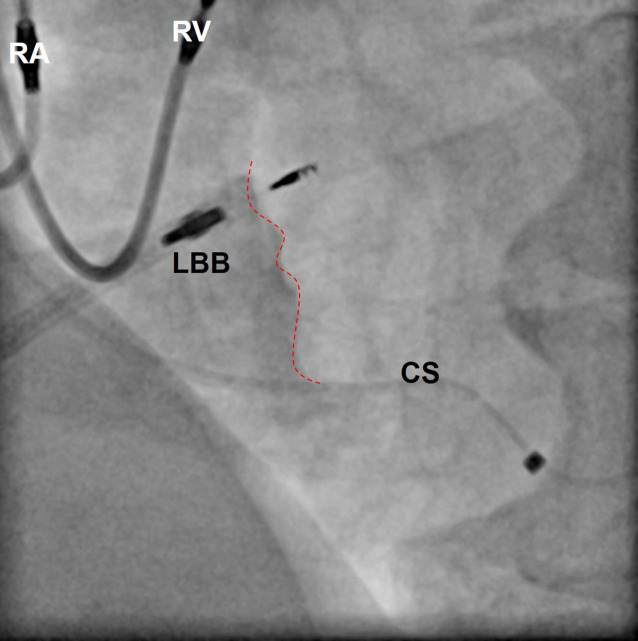
Fluoroscopy with contrast in left bundle branch (*LBB*) pacing. In this left anterior oblique view at 40°, the LBB pacing lead is visible screwed into the interventricular septum. *Dotted line*: right ventricular surface of the septum. At a depth of > 10 mm, it captured the proximal LBB. Upgrade in a patient with chronic failure of the left ventricular lead implanted via the coronary sinus (*CS*). *RA* right atrial lead at the high right atrial septum, *RV* right ventricular lead at the mid right ventricular septum

**Fig. 9:**
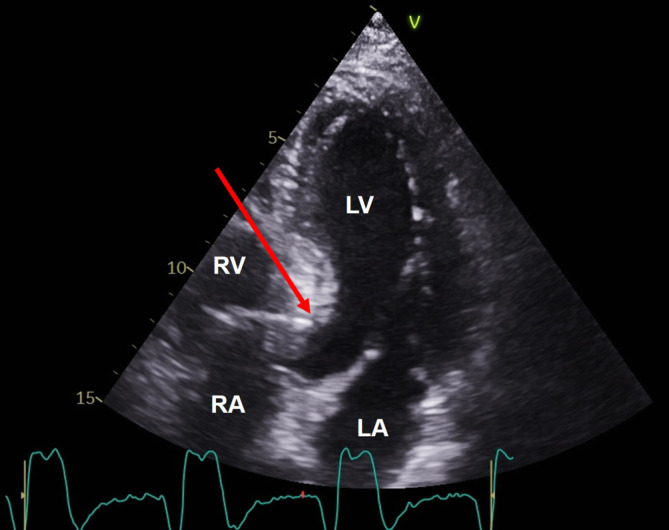
Echocardiography in left bundle branch (LBB) pacing. In the apical view, the tip of the LBB pacing lead (*arrow*) is visible just under the left ventricular endocardial surface. *LA* left atrium, *LV* left ventricle, *RA* right atrium, *RV* right ventricle

The pacing lead has to be implanted perpendicular to the right septal surface (Fig. 8) to avoid an inadvertent apical position of the lead tip and to minimize the length of transmural penetration before the left side of the interventricular septum is reached. Ideally, a paced R wave in lead V1 will appear, sometimes together with an LBB potential during sensing. This has to be checked carefully since, at this point, the lead is close to the left endocardium (Fig. 9) and should not be screwed-in further, otherwise perforation into the left ventricle can occur.

The LBB lies within the left side of the septum in a variable depth as accessed from the right side, typically > 10 mm, deeper in elderly patients (fatty infiltration), and much deeper in patients with septal hypertrophy due to hypertension, hypertrophic cardiomyopathy, or amyloidosis. In these patients, it may be difficult or even impossible to advance the lead deep enough to reach the LBB due to fibrotic, very hard tissue. Of note, the LBB is also insulated by a variable, sometimes very thick sheath of fibrotic tissue. The large variability in diameter, length, location, ramifications, and insulation of the bundles and their fascicles explains the variability of the paced QRS morphology.

## Practical conclusion

Right ventricular leads can be much better implanted if the anatomy is well known to the implanter. Only if this condition is fulfilled can the desired positions be reached and identified. Understanding of the compartments inlet, outlet, and apex, as well as myocardial structures such as tricuspid valve leaflets and their commissures, papillary muscles, septomarginal trabecularizations, and the moderator band can turn unclear obstacles into structures that can help to navigate through the RV and fix the lead at a safe site.

## Declarations

For this article no studies with human participants or animals were performed by any of the authors. All studies mentioned were in accordance with the ethical standards indicated in each case.
